# Without Borders? The Impact of Political Barriers and Land Use on the Animal Health Dynamics and Genetic Structures of Large Game Species in the Carpathian Basin and Surrounding Regions—A Systematic Review

**DOI:** 10.3390/vetsci13030302

**Published:** 2026-03-23

**Authors:** Zoltán Bagi, Renáta Knop, Camelia Tulcan, Roberta Tripon, Răducu Marinaș, Szilvia Kusza

**Affiliations:** 1 Centre for Agricultural Genomics and Biotechnology, Faculty of Agricultural and Food Sciences and Environmental Management, University of Debrecen, Egyetem tér 1, H-4032 Debrecen, Hungary; bagiz@agr.unideb.hu; 2 Department of Animal Husbandry, Institute of Animal Science, Biotechnology and Nature Conservation, Faculty of Agricultural and Food Sciences and Environmental Management, University of Debrecen, Böszörményi út 138, H-4032 Debrecen, Hungary; dr.knop.renata@agr.unideb.hu; 3 Forestry Department, Faculty of Engineering and Applied Technologies, University of Life Sciences “King Mihai I” from Timișoara, Calea Aradului 119, 300645 Timișoara, Romania; cameliatulcan@usvt.ro (C.T.); cristian.marinas@usvt.ro (R.M.); 4 Genetic Engineering Department, Faculty of Engineering and Applied Technologies, University of Life Sciences “King Mihai I” from Timișoara, Calea Aradului 119, 300645 Timișoara, Romania; roberta.tripon@usvt.ro

**Keywords:** Carpathian Basin, One Health, transboundary disease, wildlife connectivity, African swine fever, golden jackal, cervids

## Abstract

The Carpathian Basin is a coherent biogeographic unit whose wildlife populations and pathogen dynamics are increasingly reshaped by administratively fragmented governance, land-use change and linear infrastructure. This review synthesizes evidence that the permeability patterns governing host movement also structure the transboundary exchange of genes and infections, creating a connectivity substrate for conservation genetics and *One Health* risk. Focusing on wild boar (*Sus scrofa*), red deer (*Cervus elaphus*), roe deer (*Capreolus capreolus*), fallow deer (*Dama dama*) and the expanding golden jackal (*Canis aureus*), we integrate population genetic inferences with wildlife epidemiology to examine how highways, border fences and asymmetric management (e.g., supplemental, feeding practices, hunting pressure and surveillance regimes) can generate biological asymmetries across boundaries. We highlight African swine fever as an emblematic disturbance in wild boar populations, discuss cervid risks including tick-borne pathogens and chronic wasting disease (CWD) preparedness and evaluate zoonotic threats associated with carnivore expansion (e.g., *Echinococcus* spp.). We propose a Carpathian Basin-level monitoring and data-sharing architecture, coupling harmonized passive surveillance, strategic active surveillance for priority pathogens, and standardized genetic marker panels supported by interoperable metadata. A Basin-scale *One Health* approach is a pragmatic prerequisite for the coordinated prevention, early detection and resilient management of cross-border epizootics and zoonotic threats.

## Simple Summary

Wild animals move across the Carpathian Basin regardless of political borders. Their movements connect populations genetically and also connect areas through the spread of pathogens and parasites. In recent years, highways, fenced transport corridors and border barriers have reduced landscape permeability and reshaped where wildlife can cross, while neighboring countries often apply different hunting pressures, feeding practices and disease surveillance measures. Focusing on wild boar, red deer, roe deer, fallow deer and the expanding golden jackal, this review explains why these mismatches between ecological connectivity and administrative management can increase transboundary animal-health and zoonotic risks. We summarize how population genetic tools and landscape modeling can be used to map connectivity and we highlight priority threats such as African swine fever in wild boar, tick-borne hazards in deer and zoonotic parasites associated with carnivore expansion. Finally, we propose a Carpathian Basin-level *One Health* approach based on harmonized wildlife health monitoring, coordinated carcass search and reporting and cross-border sharing of genetic and epidemiological data to improve early warnings and the prevention of future outbreaks.

## 1. Introduction

Political borders are among the most conspicuous human inventions on landscapes that, ecologically, rarely end at a customs gate. Across the Carpathian Basin and its surrounding mountain arc, recent events have made this tension tangible: security-driven border infrastructure has transformed long-established wildlife movement routes into abrupt, hazardous discontinuities. Following the erection of a razor-wired fence along exposed sections of the Slovenia–Croatia border (178 km), systematic searches documented wildlife mortality and, more importantly, warned of a longer-term barrier effect that could fragment habitats and disconnect populations [1]. Comparable dynamics emerged along the Hungary–Croatia border, where monitoring of a 136 km fenced section recorded direct fence-related mortality in large ungulates and wild boar and provided field evidence of large red deer aggregations repeatedly attempting to cross the barrier [2]. These observations are not isolated anecdotes: they exemplify how political decisions, enacted as physical structures, can rapidly alter permeability in a region where ecological connectivity has historically been shaped by topography, land cover and seasonality rather than governance.

The Carpathian Basin is frequently treated as a coherent biogeographic unit arena because its basin-like lowlands are encircled by the Carpathians and connected to adjacent regions through a limited set of corridors, passes and riverine plains that channel dispersal and gene flow. Genetic reconstructions of regional history reinforce this view. Red deer populations sampled from within the Basin bear traces of dispersal and admixture consistent with post-glacial recolonization dynamics operating at the scale of the Basin rather than within single administrative units [3]. Wild boar, a species capable of rapid demographic expansion and long-distance movement, also exhibits a population genetic structure that is meaningfully interpreted at the Carpathian Basin scale [4]. Even within red deer, the Carpathians have been highlighted as a distinct phylogeographic context in which population history and contemporary mixing can be disentangled using mitochondrial and nuclear markers [5]. These studies support a pragmatic ecological claim with immediate management implications: although political borders subdivide the Basin into multiple jurisdictions, the biological units relevant to wildlife population dynamics often remain transboundary [3,4]. In the Carpathian Basin, the central lowlands act as a high-connectivity hub for host movement and human-mediated pathogen transport, while the surrounding mountain arc acts as a permeable filter. The mountains offer refuge, while the plains’ linear infrastructure creates anthropogenic bottlenecks. To understand the Basin as a functional unit, the mountain arc must be seen as a dynamic interface where ecological corridors are increasingly redirected into constrained, high-risk transit points.

Mismatch between ecological and administrative geography matters because large game species in the Carpathian Basin are both highly mobile and closely entangled with human land use. In this review, we focus on five taxa that dominate the region’s wildlife–livestock–human interface: wild boar (*Sus scrofa*), roe deer (*Capreolus capreolus*), red deer (*Cervus elaphus*), fallow deer (*Dama dama*) and the expanding golden jackal (*Canis aureus*). Their movements, whether dispersal, seasonal ranging or routine foraging, are increasingly negotiated through a landscape engineered for people. Road networks and associated fencing can simultaneously reduce some conflicts while creating new forms of fragmentation. On fenced highways in Hungary, spatial analyses of wildlife–vehicle collisions have shown that collision hotspots can still emerge where permeability is locally increased, for example at interchanges and other structural features that facilitate wildlife access to road corridors [6]. Empirical work in a red deer habitat crossed by a newly built fenced highway documented measurable changes in movement patterns and underscored the functional importance of wildlife crossings as partial mitigation of barrier effects [7]. Recent syntheses of wildlife overpass design in Hungary similarly frame fenced roads as drivers of isolation that necessitate planned permeability to sustain connectivity for large ungulates [8]. Beyond transport corridors, broader planning approaches have been proposed to reduce wildlife–vehicle collisions while recognizing that solutions must be embedded in landscape-scale design rather than treated as isolated engineering fixes [9]. The same logic underpins earlier corridor concepts such as green bridges in Austria, where wildlife corridors were explicitly justified as tools to counteract anthropogenic barriers that restrict genetic interchange between populations [10].

The argument for a *One Health* framing follows directly from these coupled realities. In the Carpathian Basin, land use does not merely set the stage for wildlife ecology; it also structures the opportunities for zoonotic pathogens and parasites to persist, amplify and spill over. Vector ecology provides a clear illustration. In south-western Slovakia, the relative density and phenology of host-seeking ticks varied between urban/suburban sites and less fragmented forest habitats, emphasizing that human-modified environments can reshape exposure risk at fine spatial scales [11]. Host communities further connect wild and domestic compartments: surveillance of haemotropic mycoplasmas and blood piroplasmids in Slovakia documented infections across domestic and wild ruminants, highlighting the permeability of pathogen transmission systems where livestock production, hunting landscapes and wildlife populations overlap [12]. Even when pathogens are not classic zoonoses, their occurrence in wildlife signals shared environmental pathways and cross-species contact structures that matter to veterinary systems. In the Slovak Carpathians, detection of *Lawsonia intracellularis* in fecal samples from multiple wild mammals illustrates that multi-host pathogen circulation can be assessed only when surveillance embraces entire communities rather than single target species [13]. For wild boar, the *One Health* rationale becomes even more explicit because food safety, livestock biosecurity and wildlife management are tightly coupled. Regional studies demonstrate sustained circulation of *Trichinella* spp. in wild boar populations, and host-diverse sylvatic cycles have been documented in the broader Carpathian region [14,15]. The *One Health* framing is essential for preventive biosecurity against emerging threats, such as Chronic Wasting Disease (CWD), in addition to active zoonoses. Although CWD has not yet taken root in the Carpathian Basin, the landscape architecture described—which includes artificial aggregations at border fences and high-density cervid ‘bottlenecks’—creates an environment that is conducive to environmental problems. The anthropogenic concentration of red and roe deer at restricted transit points [2] represents a significant vulnerability because prions persist in the soil and transmission is density-dependent. In order to stop the quick, landscape-wide “seeding” of environmental reservoirs that national-level surveillance would probably discover too late, a basin-scale preparedness strategy is therefore not just a veterinary luxury but a mechanical necessity.

If land use and host ecology are the substrates, political borders and governance determine how societies respond. Disease control and biosecurity measures can themselves become drivers of ecological change when they alter movement routes, mortality, or habitat permeability. The case of African swine fever (ASF) is emblematic because it is simultaneously a wildlife disease, an agricultural crisis and a policy trigger. Detailed investigation of a Serbian ASF outbreak in 2023 documented African swine fever virus detections in environmental samples and insects, reinforcing that transmission systems can include multiple routes beyond direct animal-to-animal contact [16]. At the human interface, a Central European survey of hunter interactions with pig farming highlighted behavioral and contact pathways that can bridge wildlife and domestic settings and therefore belong centrally within a *One Health* risk assessment [17]. Yet the very measures deployed in response to disease or security concerns, including extensive border fencing, can create ecological side effects by restricting transboundary movement, increasing local densities and potentially altering gene flow patterns over time [1,2].

Wildlife genetics offers a powerful integrative lens on this problem because it provides measurable traces of connectivity. Genetic admixture patterns in red deer from within the Carpathian Basin demonstrate that dispersal and mixing are not abstract concepts but detectable processes that have shaped populations in ways that are relevant to management units such as game reserves [3]. Similarly, wild boar genetic structure in the Basin provides baseline information against which future change, whether driven by habitat fragmentation, harvest regimes or disease control measures can be assessed [4]. The rapid recent dynamics of the golden jackal further emphasize that connectivity can change over short timeframes; evidence for hybridization signals in Carpathian Basin golden jackals, obtained through targeted genetic analyses, exemplifies how expanding mesocarnivore populations can introduce new ecological interactions and pathogen pathways into landscapes already shaped by intensive human use [18]. This review adopts a *One Health* premise: where genes move, hosts move and where hosts move, pathogens and parasites can move as well. Therefore, when permeability is constrained, both genetic and epidemiological compartmentalization may emerge [1,2,3].

Against this background, this review synthesizes evidence on how political borders, border-associated infrastructure and land-use patterns shape (i) wildlife movement and habitat connectivity, (ii) population genetic structure and diversity, and (iii) the circulation of zoonotic and veterinary-relevant pathogens and parasites. We focus on five local species—wild boar, roe deer, red deer, fallow deer and golden jackal—across the Carpathian Basin and directly adjacent regions. By integrating population genetics, landscape ecology, and wildlife epidemiology, we identify where current knowledge is robust, where it remains fragmentary and which research and monitoring priorities are most likely to strengthen transboundary *One Health* preparedness and management coherence in this politically partitioned but biologically connected region.

## 2. Literature Search and Study Selection

This systematic review was conducted in accordance with the Preferred Reporting Items for Systematic Reviews and Meta-Analyses (PRISMA) 2020 guidelines [19]. The review was designed within a *One Health* perspective, integrating evidence on population genetic structure and connectivity with wildlife health dynamics in a transboundary context. The geographic scope focused on the Carpathian Basin sensu the transboundary lowland and upland system centered on present-day Hungary, together with the immediately adjacent border landscapes of Slovakia, Ukraine, Romania, Serbia, Croatia, Slovenia, and Austria, where cross-border movement, landscape permeability or management asymmetries are relevant. The present review focused on the five focal species defined previously. Eligible studies were peer-reviewed original research articles that reported population genetic outcomes (e.g., mitochondrial DNA (mtDNA), microsatellites, single nucleotide polymorphism (SNP)-based analyses, population structure and connectivity measures), and/or wildlife health outcomes relevant to veterinary surveillance and zoonotic risk (e.g., pathogen or parasite occurrence, prevalence/serology, or related epidemiological evidence) in the target region. Studies were excluded if they did not address the focal taxa, lacked relevance to the Carpathian Basin context, did not report genetic or wildlife health outcomes pertinent to the review questions, or were not primary research (e.g., reviews, editorials). Conference-only records without sufficient methodological and results information to support extraction were also excluded. Standardized exclusion reasons applied during screening included wrong focal species, wrong geographic scope, wrong outcome domain, non-primary publication type, insufficient methodological/results detail, duplicate record or no digital object identifier (DOI) available for the retrieval workflow.

Searches were performed in PubMed, Web of Science Core Collection and Scopus. PubMed was searched on 21 February 2026, and Web of Science and Scopus were searched on 22 February 2026. Across databases, the core search logic followed the structure (focal species terms) AND (geographic terms) AND (genetics/health-related terms), with an additional AND (barrier/permeability terms) block used where relevant. Species terms included both scientific and common names of the focal taxa. Geographic terms included Carpathian Basin and country/region variants. Genetics/health-related terms covered population genetic and phylogeographic keywords, marker-related terms (e.g., microsatellite, mtDNA, SNP), and veterinary/epidemiological keywords (e.g., surveillance, pathogen, parasite, zoonosis), complemented where appropriate by targeted disease terms such as African swine fever. To capture studies explicitly addressing permeability and fragmentation, additional terms referring to borders, fences, barriers, roads/highways and landscape resistance were included. Database-specific syntax was adapted as needed, while the conceptual structure of the search was kept consistent across databases.

**Figure 1:**
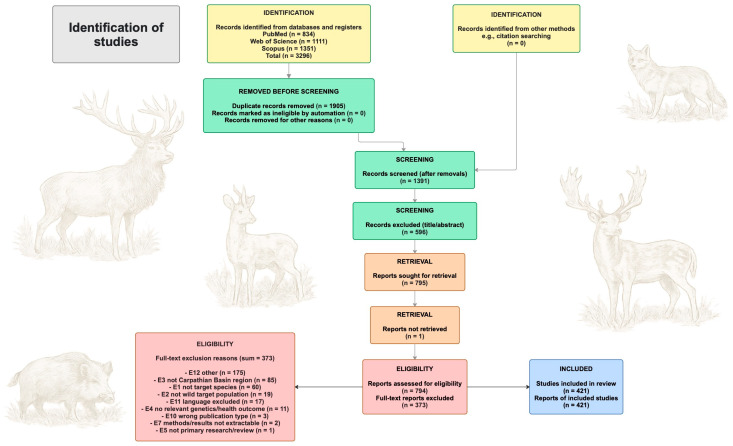
PRISMA 2020 flow diagram summarizing database searches, deduplication, screening, full-text eligibility assessment and inclusion of studies in the review.

All records were exported in native formats (PubMed .nbib, Web of Science .ciw, Scopus.ris) and managed in a reference management workflow, where duplicates were identified and merged using automated duplicate detection followed by manual verification for residual duplicates (e.g., minor title variations or differing metadata between databases). Screening proceeded in two stages. First, titles and abstracts were assessed against the predefined eligibility criteria and each record was coded as included or excluded with a standardized exclusion reason. Second, full texts were retrieved for records prioritized for full-text assessment and were evaluated for final inclusion; exclusions at this stage were also documented with standardized reasons. Full texts were obtained primarily via DOI-based resolution and institutional access, supplemented where necessary by legal open-access versions and repository copies. Records lacking a DOI could not be handled consistently within the DOI-based retrieval and tracking workflow used for full-text acquisition and were therefore excluded from further processing. We recognize that this pragmatic criterion may have underrepresented some older or regionally disseminated studies and may therefore represent a source of selection bias. Both title/abstract screening and full-text assessment were performed against the same predefined eligibility framework. The record screening, full-text assessment and inclusion process is summarized in the PRISMA 2020 flow diagram (Figure 1) (Table S1).

Data were extracted using a standardized template capturing bibliographic information, species, study area, sampling design, and the main genetic and/or veterinary outcomes relevant to cross-border connectivity and health dynamics. Title/abstract screening and full-text eligibility assessment were performed by Z.B.; any uncertainties were resolved through discussion with S.K. We did not perform independent dual screening, which should be considered a methodological limitation and a potential source of selection bias. Data extraction was performed by Z.B. and cross-checked for consistency by S.K. and R.K. For genetics-focused studies, extraction recorded marker type, analytical approach, and key findings on population structure and gene flow; for health-focused studies, it recorded the agent investigated, diagnostic approach, and reported occurrence or prevalence metrics where available. Evidence referring to barriers or filters (e.g., border fences, highways, land-use discontinuities) and management context (e.g., fenced enclosures, hunting pressure, surveillance intensity) was captured when reported. Because study designs and outcomes were heterogeneous across wildlife genetics, landscape studies, and veterinary surveillance, a quantitative meta-analysis was not pursued. We also did not apply a formal risk-of-bias tool; instead, we provide a qualitative appraisal emphasizing methodological transparency and relevance to the review questions. Results were synthesized narratively, organized by focal species, and integrated across themes to support a One Health interpretation of how natural and anthropogenic boundaries shape connectivity and wildlife health dynamics in the Carpathian Basin region. This review was not registered, and no prospective protocol registration was performed, because the work was conceived as an interdisciplinary evidence synthesis across heterogeneous observational wildlife-genetics and wildlife-health literature rather than as an intervention-focused review developed within a registry workflow. To support transparency, the review questions, eligibility criteria, search strategy and extraction framework were defined a priori and are reported in accordance with PRISMA 2020.

## 3. Conceptual and Methodological Framework: Linking Gene Flow to Pathogen Spread

A central premise of this review is that the Carpathian Basin functions biologically as a connected system, even when it is administratively partitioned. This premise is not merely rhetorical; it is measurable. In population genetics, connectivity is inferred from the spatial distribution of genetic variation produced by dispersal and reproduction. In epidemiology, connectivity is expressed through the movement- and contact-mediated spread of infectious agents across host populations. Although these processes operate on different timescales, their shared dependency on host movement creates a powerful analogy for transboundary inference: where individuals routinely cross permeable landscape features, both alleles and infections are more likely to cross as well, whereas barriers that reduce movement tend to increase genetic differentiation and can also slow or re-route pathogen diffusion.

Population genetic markers provide the empirical backbone for quantifying wildlife connectivity because they capture the cumulative outcome of dispersal, mating and demographic history. Microsatellites remain widely used in large-mammal systems because their high allelic diversity yields sensitivity to recent structure and admixture, particularly at the spatial scales relevant to management. In red deer from the Carpathian Basin, multi-locus microsatellite data have been used to estimate heterozygosity and allelic richness, to identify spatially coherent clusters and to apply assignment tests that detect recent movement among reserves, thereby translating allele frequencies into evidence for contemporary dispersal and admixture [3]. Earlier work on Carpathian red deer similarly combined mitochondrial control-region sequences with nuclear microsatellites to resolve phylogeographic structure, private alleles and genetic distances across the Romanian Carpathians and adjacent Serbian study areas, illustrating how marker choice can separate deeper lineage history from more recent connectivity signals [5]. For wild boar, Carpathian Basin-scale inference based on multi-locus genotypes and Bayesian clustering approaches has likewise been used to describe population genetic structure in a species whose mobility and demographic elasticity make it a particularly informative connectivity tracer for the region [4]. At a more applied level, standardized microsatellite panels have been optimized specifically for individual identification and parentage assignment in wild boar populations including samples from Hungary, demonstrating that the same marker systems can quantify both broad-scale structure and fine-scale kinship and recruitment processes that underpin realized gene flow [20].

In parallel, SNP-based approaches increasingly enable higher-resolution connectivity inference and facilitate explicit integration with landscape predictors. Genome resources such as the red deer reference assembly CerEla1.0, which reports millions of SNPs and frames their utility for genome-wide analyses, lower the barrier to moving from locus-limited summaries toward genome-enabled models of structure, admixture and adaptation [21]. In wild boar, SNP genotyping has already been applied at an urban–peri-urban interface in Budapest, where SNP-based differentiation and fixation index (F_ST) estimates revealed subpopulation structuring across the Danube and highlighted founder effects and inbreeding within an urban population, illustrating how contemporary barriers and corridors can be detected even within a single metropolitan landscape [22]. SNP datasets are also particularly valuable for identifying contact zones between wild and domestic compartments, which is epidemiologically consequential in systems where livestock–wildlife spillover is mediated by shared space and human-managed interfaces. For example, European-wide SNP analyses have mapped hotspots of pig–wild boar hybridization, including signals in parts of Southeast and Central Europe, indicating where sustained contact is sufficient to leave a genomic imprint and, by extension, where pathogen exchange opportunities between domestic and wild hosts may be elevated [23,24]. Recent range-wide whole-genome resequencing in red deer further underscores that connectivity is marker- and timescale-dependent: sex chromosomes can retain demographic and phylogeographic signals differently from autosomes, with direct implications for how recent fragmentation is distinguished from deeper historical structure [25]. For transboundary *One Health* inference, this matters because genomic compartment choice can influence which movement processes are emphasized (e.g., long-term dispersal versus contemporary permeability) and thus how confidently gene-flow patterns can be interpreted as proxies for pathogen spread.

Landscape ecological modelling supplies the mechanistic bridge that makes the gene–pathogen analogy actionable. Landscape genetics replaces purely geographic distance with effective distance derived from hypothesized resistance surfaces, allowing researchers to test whether genetic differentiation aligns more closely with habitat permeability than with straight-line distance. In the southeastern Carpathians, circuit-theory modelling implemented through Circuitscape has been used to generate multispecies connectivity surfaces and corridor maps for red deer, wild boar and roe deer (among others), explicitly targeting the mitigation of linear infrastructure impacts and identifying spatial bottlenecks where permeability is most critical [26]. Complementary spatial methods can diagnose where movement is most concentrated or disrupted, even when genetics is not directly measured. Kernel density estimation of wildlife–vehicle collision locations in the southeastern Carpathians has been used to delineate hotspots involving the same focal ungulate species, providing an independent, data-driven proxy for where movement intersects with anthropogenic infrastructure and therefore where landscape pinch points may influence both dispersal and contact structure [9]. These modelling approaches matter for *One Health* because the same corridor networks and bottlenecks that structure dispersal also structure encounter rates, density-dependent contacts and human-mediated interfaces (such as carcass removal, supplemental feeding, hunting intensity), each of which can accelerate, redirect, or locally amplify infection dynamics.

The epidemiological side of the analogy becomes especially transparent when infectious agents are analysed with the same population-structuring logic used for hosts. Risk assessment models for African swine fever explicitly treat wild boar-mediated spread as a transboundary process in which introduction probability and subsequent spread depend on spatial proximity to infected areas and the movement ecology of the host [27,28]. At the policy-relevant scale, the European Food Safety Authority (EFSA) assessment for south-eastern Europe concluded that the probability of ASF spread within the region of concern within one year after introduction was very high, emphasizing that risk is regional and not confined to the jurisdiction where detection first occurs [29]. Empirically, spatiotemporal analyses of ASF detections in wild boar in Serbia have further demonstrated how outbreak intensity and spatial clustering evolve through time, offering a statistical description of diffusion patterns that can be compared directly against predicted connectivity corridors or resistance-mediated effective distances [30]. When pathogen genomics is added, the parallel to landscape genetics becomes even stronger: phylogeographic reconstruction is, in essence, connectivity inference performed on pathogen lineages. Whole-genome sequencing and SNP-based phylogenetics have been used to resolve the spatial structure of *Brucella suis* biovar 2 across wildlife and domestic swine, revealing clade structure and geographic signatures that were previously obscured by high nucleotide identity and thus enabling more explicit hypotheses about cross-border maintenance and spread [31,32]. Similarly, genomic analyses have been used to document cross-border transmission of *Salmonella choleraesuis* var. *Kunzendorf* across European pig and wild boar systems, illustrating that pathogen lineages can track transboundary connectivity even when surveillance and control are nationally organized [33].

## 4. Cervidae in the Carpathian Basin: Fragmentation, Connectivity and Health Risks

Cervids are among the clearest biological readouts of how political borders and land-use change translate into *One Health* outcomes in the Carpathian Basin. Red deer and roe deer, in particular, occupy large home ranges or landscapes of repeated seasonal movement, interact with managed habitats (forests, agricultural mosaics, peri-urban edges) and are embedded in hunting systems that routinely cross administrative boundaries through shared ecological corridors. Fallow deer, by contrast, is often embedded in more intensively managed settings, including fenced estates and game parks, where density and contact structures can diverge strongly from those of free-ranging populations. Across all three species, fragmentation is therefore not a purely conservation concern: it can reshape contact rates and movement networks that underpin pathogen persistence, spillover opportunities and over longer time horizons, the distribution of genetic diversity.

### 4.1. Red Deer (Cervus elaphus): Linear Barriers, Connectivity Loss and CWD Preparedness

Red deer exemplifies the central tension of this review: a biologically connected system managed through politically partitioned decision-making. Genetic data from Hungary, representing seven game reserves within the Carpathian Basin, depict a species with high diversity and substantial contemporary mixing. In a microsatellite-based analysis of 303 individuals, Frank et al. [3] reported consistently high within-population diversity (allelic richness 4.99–7.01; observed heterozygosity 0.729–0.800) and a spatial pattern in which south-western and north-eastern groups formed two more separated clusters, while populations in between were strongly admixed. Importantly for connectivity, assignment tests indicated that nearly one third of individuals (29.4%) were detected outside their inferred population of origin, a direct signature of frequent dispersal at management-relevant scales [3]. This is precisely the kind of genetic evidence that complicates purely national or reserve-based units and supports a Carpathian Basin framing: movement is common enough to blur boundaries under typical conditions.

Yet the same dataset also hints at how anthropogenic linear infrastructure can convert movement potential into realized restriction. Frank et al. [3] explicitly note that the most distant regions in their Hungarian sampling design are separated by historical landscape features and, later, by industrialisation and main rail- and highways, underscoring that connectivity is not simply a function of distance but of permeability. Field-based movement studies reinforce how quickly a fenced motorway can impose functional discontinuity. In a red deer landscape bisected by a newly built fenced highway, Ballók et al. [7] observed that, after completion, only 5.9% of the original track counts were recorded at crossings and that deer preferentially used larger crossing structures, illustrating both a strong barrier effect and the partial mitigation potential of appropriately designed passages. Even when roads are fully fenced, collision patterns show that permeability is not eliminated but redistributed; on Hungarian fenced highways, interchanges emerged as focal collision hotspots, including substantial roe deer mortality, highlighting where animals most often encounter the infrastructure boundary [6]. At broader administrative scales, modelling and empirical analyses from Central Europe similarly emphasize that roads and their associated fencing restructure wildlife space use and can shift demographic patterns across districts [34]. The implication for red deer genetics is not that every motorway immediately produces measurable divergence, but that the conditions that sustain gene flow repeated safe passage and inter-population mating, can be selectively eroded and that the time-lag between functional fragmentation and detectable genetic fragmentation should not be used as a rationale for inaction.

Border fences sharpen this problem because they are designed to reduce permeability, often abruptly, across landscapes that historically functioned as movement corridors. On the Slovenia–Croatia border, a razor-wired fence erected in late 2015 was associated with documented direct mortality of 21 ungulates over a 10 months (13 red deer, 8 roe deer), while the authors explicitly raised the longer-term ecological concern of barrier-driven fragmentation [1]. Along the Hungary–Croatia border, fence monitoring similarly documented direct mortality and provided field evidence of repeated crossing attempts and aggregation behaviour in red deer [2]. These observations are not genetic studies, but they are mechanistically decisive: if crossing probability declines at a state boundary, the opportunity for mating-mediated gene flow declines as well. A critical gap in the Carpathian Basin is therefore the limited availability of before-and-after genetic monitoring explicitly designed to quantify barrier effects of border infrastructure. In the absence of such designs, the region risks discovering fragmentation only after its genetic signature becomes difficult to reverse. Instead of blocking genes, these physical barriers concentrate individuals. Pathogen shedding is high at fence-line dead-ends due to this aggregation behavior. This behavior causes concentrated environmental seeding of prions at anthropogenic bottlenecks in CWD, creating long-term “hot zones” that persist after connectivity is restored or the fence is removed.

From a *One Health* perspective, the same permeability changes matter because red deer are not merely carriers of genes; they are also potential carriers of pathogens and parasites whose spread is mediated by movement and contact. While much of the Central European surveillance discourse has focused on ASF in wild boar, emerging cervid diseases deserve proactive attention because they can arrive quietly, exploit existing connectivity and become costly to control once established. Chronic wasting disease, a fatal prion disease of cervids, has been detected in Europe since 2016 [35] and has prompted EFSA to propose monitoring and control measures explicitly framed around the movement of live cervids and transboundary prevention. In EFSA’s summary of the multi-country monitoring programme (2017–2022), cases were detected in reindeer, moose and red deer, confirming that the disease has already involved a species central to Carpathian Basin wildlife management. Although CWD detections have, to date, been concentrated in northern Europe, the key lesson for the Carpathian Basin is that preparedness must be regional rather than national. Monitoring should be coordinated along likely entry pathways (animal movements, trade-related translocations and high-connectivity corridors) and integrated with an understanding of where permeability is already being altered by infrastructure. Without such integration, border fences may create an illusion of protection while simultaneously concentrating cervid movements and contacts at specific leak points (interchanges, river crossings, unfenced segments) where both genetic exchange and pathogen exchange remain possible.

### 4.2. Roe Deer (Capreolus capreolus): Fine-Scale Genetic Structure and Tick-Borne Disease Risk

Roe deer differ from red deer in ecology in ways that matter for both genetics and disease. Their generally stronger site fidelity and smaller spatial scale of routine movement tend to produce finer-grained population structure, especially in heterogeneous landscapes. Classic genetic work across Central Europe, based on 40 isoenzyme loci, described roe deer as among the more genetically variable deer species studied while also showing that meaningful differentiation can occur between demes, with roughly 10% of total diversity attributable to between-population differences [36]. Most strikingly for the theme of political barriers, Hartl et al. [36] interpreted distinct differentiation in Hungary—relative to Austrian and Swiss provenances—as plausibly linked to completely fenced borders between Austria and its eastern neighbours, an early genetic echo of how border infrastructure can disrupt gene flow. Contemporary microsatellite analyses from adjacent regions similarly underscore that roe deer structure can reflect management history and connectivity rather than simple geographic distance. In Slovenia, roe deer females sampled across the Alps–Dinaric contact zone exhibited spatially structured genetic diversity and admixture patterns consistent with strong philopatry but non-negligible gene exchange among areas and the authors also reported heterozygosity–fitness relationships that imply demographic consequences of genetic variation [37]. In practical terms, roe deer genetics often maps onto local neighbourhoods, making the species an informative sentinel for identifying where fragmentation is already expressed at the scale of counties, districts, or border-adjacent landscapes.

This matters directly for tick-borne diseases because, in Central Europe, the epidemiology of Lyme borreliosis and tick-borne encephalitis (TBE) is strongly focal, reflecting the ecology of *Ixodes* ticks, microclimate and host community composition. Roe deer are important maintenance hosts for ticks and can influence pathogen communities without necessarily being competent reservoir hosts for every agent of concern. At the same time, small mammals may serve as important reservoir hosts for several tick-borne pathogens and broader community composition, including predator–prey dynamics, may influence both pathogen amplification and vector population dynamics. Detailed field data from south-western Slovakia show that the density and phenology of host-seeking *Ixodes ricinus* can vary substantially among habitat types, including urban/suburban versus more continuous forest settings, indicating that land use can reshape exposure risk at fine spatial scales [11]. Surveillance across free-living ungulates in the same region detected diverse tick-borne microorganisms in tissues and engorged ticks collected from roe deer, red deer, fallow deer, wild boar and mouflon, reinforcing that ungulates contribute to multi-pathogen transmission networks rather than to single-agent cycles [38]. At the interface between host identity and pathogen diversity, work in Central Europe has shown that roe deer presence can be associated with shifts in the occurrence of *Anaplasma phagocytophilum* ecotypes in questing *I. ricinus* across habitat types [39]. This implies that local ungulate community structure may shape not only tick abundance but also the genetic/ecological composition of circulating pathogens.

The relationship between deer abundance and human disease incidence is therefore not straightforward and this is precisely where a critical *One Health* interpretation is needed. In Slovenia, temporal and spatial analysis found a correlation between red deer abundance and human TBE incidence, while a comparable correlation was not confirmed for roe deer, likely because roe deer density was already above a threshold where further increases did not measurably change tick reproductive host availability [40]. This result is a useful caution against simplistic narratives that equate more deer with more disease. It also highlights why roe deer, with their local genetic structuring, are particularly relevant: interventions that change roe deer density or movement at local scales may alter tick ecology and pathogen exposure in ways spatially heterogeneous, producing high-risk foci rather than uniform risk.

Linear infrastructure can intensify this heterogeneity. In Hungary, a fenced freeway was shown to be associated with differences in tick diversity and *Ixodes*-borne pathogen prevalence between its two sides [41]. The authors further argued that forested resting areas adjacent to the freeway may become Lyme disease hotspots, consistent with barrier-induced host/vector segregation and concentrated activity in accessible patches. Serological approaches developed and applied to game animals in Slovakia further demonstrate that, even when pathogen detection is challenging, population-level exposure patterns can be measured and compared across host species and landscapes [42]. The emerging methodological opportunity for the Carpathian Basin is therefore integrative: Roe deer population genetics can delineate the movement neighbourhoods that underpin contact structures, while tick ecology and pathogen surveillance can map where those neighbourhoods translate into higher zoonotic hazard. What remains underdeveloped is the explicit coupling of these datasets testing whether genetically inferred connectivity surfaces predict the spatial continuity (or fragmentation) of pathogen lineages or exposure patterns, particularly across borders and across major road networks.

### 4.3. Fallow Deer (Dama dama): Enclosure Management, Escapes and Mycobacterial Risk

Fallow deer occupies a distinctive niche in the Carpathian Basin’s cervid assemblage because its distribution and demography are often more strongly shaped by human management than those of native cervids. This has two major *One Health* consequences. First, enclosure-based management can elevate contact rates, concentrate fecal contamination and increase opportunities for pathogens with environmental stages or intermediate hosts. Second, managed populations can be genetically narrowed and demographically structured in ways that complicate both conservation genetics and forensic attribution. Recent Hungarian datasets clearly illustrate the genetic narrowing component. Sequencing a 945 bp segment of the mitochondrial control region in 138 individuals from five populations detected only four haplotypes (one novel) and low mtDNA diversity (Hd = 0.565; π = 0.002) [43,44]. These values are comparable to low diversity reported elsewhere and are consistent with a managed population history. Marker development for fallow deer genetics and forensic applications, including microsatellite panel selection, further reflects the practical need to manage a high-value game species whose population structure is not purely natural [45].

Enclosure epidemiology is well illustrated by a Danube-adjacent case study from Serbia, where *Fascioloides magna* infection was measured and managed in a fenced fallow deer population. In that system, infection was treated through triclabendazole-medicated feed delivered at baiting stations and the authors argued that effective control required not only pharmacotherapy but also habitat interventions to reduce contact with the snail intermediate host, underscoring how semi-captive management links veterinary intervention, habitat manipulation and transmission ecology [46]. Crucially for this review’s transboundary framing, the same study interpreted the presence of *F. magna* as likely linked to natural spread across the Danube from Hungary and Croatia, demonstrating how river corridors can sustain cross-border parasite movement even when host populations are locally enclosed [46]. Parasite population genetics adds weight to the idea that foci along the Danube are connected: mitochondrial analyses of fascioloidosis in Danube and Italian foci highlight spatial structure in parasite lineages consistent with regionally connected transmission systems rather than isolated national problems [47]. In parallel, microbiological surveys from hunting reserves in western Romania show that fallow deer can host potentially pathogenic bacterial communities, reinforcing that managed cervid populations should be evaluated as components of broader veterinary risk landscapes, not as isolated game resources [48].

The question of bovine tuberculosis (bTB) and related mycobacterial hazards is particularly salient in managed cervids because detection, persistence and control are sensitive to density, husbandry and wildlife–livestock interfaces. In Slovenia, a survey of apparently healthy wild game reported mycobacterial isolation in 11.8% of tested animals and included detections in cervids such as red deer, roe deer and fallow deer, illustrating that mycobacteria circulate in wildlife even where a country is officially bTB-free and highlighting the surveillance value of wildlife sampling [49]. A multi-country Central European assessment (Croatia, the Czech Republic, Hungary, Poland, Slovakia and Slovenia) documented *Mycobacterium bovis* infection in multiple non-cattle species and included both wild and farmed red deer, as well as a captive animal bred in a game park [50]. This shows that bTB risk extends beyond free-ranging wildlife and that managed enclosures may be part of the same epidemiological network. Even when isolates are non-tuberculous, their diversity in farmed and wild animals can complicate diagnostics and risk interpretation; Hungarian work on non-avium nontuberculous mycobacteria documented wide genetic diversity and novel strains across farmed and wild hosts, underlining that mycobacterial ecology should be treated as a veterinary landscape attribute rather than a farm-only problem [51].

A critical point for the Carpathian Basin is that enclosure systems create asymmetric management conditions across borders. A fenced estate on one side of a boundary can act as a density amplifier and a pathogen reservoir, while a free-ranging population on the other side may experience lower density but higher exposure at shared corridors or at escape/ingress interfaces where fences fail, or animals are translocated. Despite the intuitive plausibility of escape-mediated risk, empirical quantification is rare and this represents a priority gap: the region needs standardized reporting and genetic assignment approaches that can detect spillover of individuals from managed populations into surrounding landscapes, paired with health surveillance targeted at pathogens where density dependence and environmental persistence make enclosures particularly consequential.

### 4.4. Synthesis and Research Priorities for Cervids

Across cervids, the *One Health* challenge in the Carpathian basin is not a lack of tools but a lack of integration at transboundary scales. Red deer genetics demonstrates substantial dispersal and admixture under typical conditions [3], while road and fence case studies show that permeability can be reduced rapidly, redistributing movement into fewer crossings and potentially creating time-lagged genetic fragmentation [1,2,7]. Roe deer genetics, in turn, offers fine-scale sensitivity to local fragmentation [36,37] and the tick-borne disease literature demonstrates that land use and infrastructure can produce focal zoonotic hazard that does not track host abundance linearly [40,41]. Fallow deer highlights how human management can narrow genetic diversity and amplify transmission in semi-captive systems with cross-border linkages sustained by river corridors and shared landscapes [44,46]. The strategic research agenda that emerges is therefore coherent: coordinated cross-border genetic monitoring (before-and-after designs around major barriers), coupled host–vector–pathogen surveillance in border-adjacent landscapes and explicit inclusion of managed cervid systems (game parks, fenced estates) as epidemiological nodes rather than private exceptions. Finally, the emergence of CWD in Europe underscores that cervid health preparedness must be framed as regional biosecurity, not as a set of national checklists [35].

## 5. Omnivores and Predators: Transboundary Epidemiology in Wild Boar and Golden Jackal

### 5.1. Wild Boar (Sus scrofa): ASF Dynamics, Barriers and Potential Genetic Consequences

Among the focal species in this review, wild boar occupies a unique position at the nexus of landscape connectivity, agricultural biosecurity and governance. Its ecological success in the Carpathian Basin is rooted in demographic plasticity, broad habitat tolerance and high mobility, characteristics that simultaneously facilitate gene flow and create an efficient host–vector for pathogens across administrative borders. Baseline population genetic evidence from the Basin underscores this duality. Using 13 microsatellite loci and a large sample set (*n* = 486), Mihalik et al. [4] reported high allelic richness (4–14 alleles per locus) and inferred two weakly differentiated subpopulations (F_ST = 0.03), while also concluding that the absence of obvious physical barriers between these groups makes contemporary landscape permeability a key context for interpreting the observed structure. In other words, the prevailing genetic signal is consistent with substantial movement and admixture operating across management-relevant spatial scales, with only shallow differentiation emerging in the absence of strong barriers [4]. Evidence from Budapest further illustrates how rapidly permeability constraints can become legible in boar genomes when movement is channelled or curtailed. SNP analyses differentiated wild boar sampled from the western and eastern banks of the Danube and detected a discrete urban subpopulation, implying that rivers and dense urban matrices can impose effective barriers and generate measurable structure even within a relatively small geographic footprint [22]. These findings matter for ASF because they indicate that connectivity is sufficiently strong to support rapid pathogen spread under typical conditions, but also sufficiently sensitive to barriers that control measures may feed back into the evolutionary and demographic system they intend to manage.

ASF is, in effect, a transboundary ecological disturbance layered onto an already fragmented governance landscape. In south-eastern Europe, EFSA’s risk assessment explicitly treated wild boar as a central driver of introduction and spread and highlighted how prevention and preparedness are inseparable from hunting practices, feeding and baiting regimes and the existence of fenced hunting estates that can maintain elevated carrying capacity [29]. The importance of this point becomes clear when outbreaks are examined in their local socio-ecological setting. Serbia’s first confirmed ASF detection in domestic pigs in 2019 [52] was followed by sustained emergence in both domestic and wild populations, and subsequent analyses framed the epidemic as a continuing, multi-pathway challenge shaped by biosecurity gaps and structural vulnerabilities in production systems [53]. In domestic pigs, transmission pattern analyses have further emphasized that outbreaks are not simply random events but reflect recurrent contact structures that need to be targeted through surveillance and control rather than assumed to be self-limiting [54]. Wild boar dynamics in Serbia add the spatial dimension: a spatiotemporal analysis covering 2020–2024 documented the evolution of case clustering through time, reinforcing that wild boar infection is best understood as a landscape process rather than as a set of isolated administrative events [30]. Genomic and regional evidence of endemic circulation across borders, such as the reported circulation of a defined ASFV cluster in Serbia and Bosnia and Herzegovina, further underlines that political borders rarely correspond to epidemiological boundaries in a mobile host system [55].

A critical methodological and practical insight emerging from the Central and south-eastern European experience is that ASF persistence is disproportionately associated with carcass-mediated transmission, particularly in wild boar. This is precisely why early detection strategies increasingly prioritise the efficient localization and removal of infected carcasses. Allepuz et al. [56] presented a structured approach for targeting carcass search to improve early detection, an operational advance that is especially relevant in heterogeneous landscapes where detection probability and search efficiency vary sharply across habitat types and seasons. The logic is straightforward and aligns tightly with the genes and pathogens follow movement premise: if connectivity is high, then rapid detection and removal must be spatially optimized along likely diffusion pathways, rather than applied evenly or purely within administrative boundaries [56]. At the same time, Serbian field data caution against underestimating environmental persistence and indirect routes. During a 2023 outbreak, ASFV detections in environmental samples and insects were reported, supporting a broader view in which contaminated substrates and potential mechanical vectors may contribute to local transmission dynamics, particularly when carcass presence is prolonged and sanitation is incomplete [16].

The most direct point of contact between ASF control and the landscape genetics framing of this review concerns physical barriers and enclosure-based management. While border fencing is often discussed in the context of security infrastructure, fences and enclosures are also embedded in wildlife management systems, including fenced hunting estates and enclosed hunting grounds. EFSA explicitly noted fenced hunting estates as a governance-relevant feature influencing wild boar ecology through year-round feeding and altered carrying capacity [29]. In Serbia, the epidemiological implications of enclosure were brought into sharp relief by the detailed report of an ASF outbreak in an enclosed wild boar hunting ground near the Serbian–Romanian border. Prodanov-Radulović et al. [57] described an outbreak in which 149 carcasses were detected across open and enclosed parts of the hunting ground, with 99 laboratory-confirmed ASF-positive carcasses and framed the event as the first documented outbreak in an enclosed wild boar population in Serbia. This case is diagnostically and conceptually important because enclosure can simultaneously reduce outward spread and increase local transmission intensity by concentrating animals, increasing contact rates and prolonging infectious carcass availability if carcass search and removal is incomplete [57]. It also highlights a less discussed evolutionary consequence: repeated enclosure, intensified culling and density manipulation can amplify genetic drift, alter age–sex structure and potentially shift effective dispersal patterns over time, thereby changing the very connectivity field that was originally inferred from baseline genetic datasets such as those presented for the Carpathian Basin [4]. In the longer run, control infrastructure can therefore become an evolutionary force, especially if fences, enclosures and hunting pressure impose consistent, spatially structured selection and demographic bottlenecks across multiple generations.

Border fences erected for non-disease reasons further complicate this picture because they can fragment movement networks and concentrate crossings into fewer leak points. Evidence from the Hungary–Croatia border fence documented fence-related mortality across several ungulate species, including wild boar and explicitly framed the fence as an obstruction to transboundary management and movement [2]. Although mortality counts are not genetic metrics, they indicate reduced permeability and altered movement behavior at the boundary, precisely the conditions under which genetic differentiation and epidemiological compartmentalization can emerge [2]. Earlier evidence from the Slovenia–Croatia border similarly warned that even if immediate mortality is modest, the long-term barrier effect may disconnect populations and constrain gene flow [1]. For ASF, this creates a management paradox: fences may be perceived as reducing transboundary spread risk, yet they may also increase local density, redirect movement and shape contact networks in ways that could locally intensify transmission or complicate carcass detection and removal. The implication for the Carpathian Basin is that ASF control should be evaluated not only as an epidemiological intervention but also as a landscape intervention whose ecological and genetic externalities must be anticipated and monitored.

Finally, ASF illustrates why *One Health* in this system cannot be reduced to wild boar versus domestic pigs. Hunter behavior and the hunting–farming interface constitute a persistent bridge between wildlife and livestock systems. A Central European survey of wild boar hunters documented frequent, policy-relevant interaction pathways with pig farming that can plausibly facilitate pathogen transfer unless explicitly managed through biosecurity training and harmonized protocols [17]. Experience from Romania underscores the complementary point from the domestic side; during 2019, a large, matched case–control study of Romanian pig farms identified ASF risk factors in a context where domestic outbreaks became widespread, reinforcing that local biosecurity and proximity-driven risk can dominate transmission in certain phases of an epidemic [58]. Even methodological work from Romanian hunting zones, such as the report of false-positive polymerase chain reaction (PCR) results for African swine fever virus (ASFV) detection in wild boars, is instructive because it emphasizes that surveillance reliability and laboratory interpretation are themselves critical elements of response capacity [59].

### 5.2. Golden Jackal (Canis aureus): Range Expansion, Population Genetics and Zoonotic Risk

The golden jackal’s rapid expansion across the Carpathian Basin and into adjacent northern regions represents a second, structurally different test of the review’s core argument. Unlike ASF, which involves a pathogen moving through a host network, golden jackal expansion reflects a change in the host network itself, reconfiguring predation pressure, scavenging dynamics and zoonotic interfaces across borders. Romania provides one of the clearest empirical narratives of this process. Using official data from more than 2100 hunting grounds, Popovici et al. [60] quantified a 31.65-fold increase in estimated golden jackal numbers between 2004 and 2025 (from 1291 to 40,861), accompanied by spatial spread from Danube-bordering counties to widespread presence across lowland and hilly regions, reaching the northern national borders. This is not simply a demographic story; it is also a governance story, because hunting-ground-based monitoring and reporting frameworks effectively operationalize the species as a managed entity whose abundance is tracked and acted upon through national systems [60]. The same dataset also highlights why expansion is intrinsically transboundary. The Danube region appears repeatedly as a spatial anchor in early colonization narratives, consistent with the riverine–floodplain mosaic functioning as both a corridor and a resource subsidy [60]. It also suggests that once a critical mass is reached, dispersal and establishment can proceed rapidly across broad parts of the Carpathian Basin.

Genetic evidence helps explain why this expansion can be fast while still leaving a detectable demographic signature. A multi-country analysis of golden jackals spanning Bulgaria, Serbia, Croatia (Dalmatia and Slavonia) and the expanding north-western range in Italy used mtDNA control region sequencing and microsatellite genotyping to infer both origin and structure. However, the mtDNA control region was monomorphic (a single haplotype across sampled populations), while microsatellites showed intermediate heterozygosity, with observed heterozygosity (Ho = 0.47) close to expected heterozygosity (He = 0.51), and significant population subdivision (h_ST = 0.07), a heterozygosity-based index of genetic differentiation among populations. Assignment and gene-flow analyses suggested that golden jackals colonizing Italy had admixed origins and that dispersal proceeded via a stepping-stone process rather than by simple first-generation migration [61]. This pattern is consistent with repeated range-edge colonization events in which long-distance dispersal is followed by local establishment and secondary dispersal, producing both spatial structure and admixture signals in the expanding zone. Importantly for *One Health*, such stepping-stone expansion implies that pathogen communities and zoonotic hazards can be redistributed in a similarly staged fashion, with newly established populations potentially sampling, filtering and amplifying parasite assemblages as they move into new landscapes.

Functional genetic data point to an additional layer: expansion may be shaped not only by demography but also by selection in novel pathogen landscapes. A study focusing on the major histocompatibility complex (MHC) class II dog leukocyte antigen (DLA) DQA1 locus across Bulgaria, Serbia and Hungary detected only three alleles, interpreted as consistent with a historical demographic bottleneck, yet also found evidence of positive selection at specific codons and suggested that allelic variation may reflect adaptation to spatially varying pathogenic environments. The same work reported associations between genotype occurrence and ambient temperature and suggested allele effects on yearling body mass index, implying potential fitness consequences relevant to expansion dynamics [62]. In practical terms, this means that golden jackal expansion is unlikely to be a purely neutral demographic wave; it may also be a moving front of host immunogenetic adaptation that interacts with local parasite and pathogen communities. Such dynamics complicate any assumption that expansion equals homogenisation in disease risk: expanding hosts can carry new hazards, but they can also experience new selective regimes that reshape susceptibility, shedding and host competence over time [62].

Movement ecology provides the mechanistic bridge from genetics to management. In south-western Hungary, GPS telemetry of 45 collared golden jackals over two years documented substantial variability in space use and distinguished resident from irruptive/nomadic individuals [63]. Among residents, the mean 95% kernel home range was ~14.4 km^2^, with strong individual, sex and age effects and frequent home-range shifts, especially in juveniles. This type of movement behavior is precisely what makes national management assumptions fragile: dispersers and roaming individuals are the vehicles of transboundary spread and they are also the individuals most likely to encounter anthropogenic food sources, livestock, carcass disposal sites and peri-urban edges, interfaces where zoonotic transmission risk is often elevated.

The zoonotic dimension is not hypothetical. Several studies from the region document that golden jackals are embedded in parasite transmission networks with direct public health relevance, particularly in areas recognized as hotspots of multilocular echinococcosis [64,65]. In Vojvodina, Serbia, intestinal helminth screening of 64 golden jackals reported that 57.8% were infected with at least one helminth species and detected *Echinococcus multilocularis* in 14.1% of examined individuals, explicitly framing the region as a hotspot context [66]. Evidence from the western Balkan region further supports that *E. multilocularis* is not confined to a single host or habitat type. In a study of 391 animals from south-western Hungary, overall prevalence exceeded 18%, with higher prevalence in golden jackals (21.1%) than in red foxes (15.2%), and landscape modelling identified wetlands and precipitation-related variables as significant drivers of local and global prevalence patterns [67]. Mechanistic work comparing parasite egg production between foxes and golden jackals adds an important nuance [68]. Although the proportion of worms producing mature eggs was lower in golden jackals than in foxes, the proportion of fertile worms and the mean egg production among egg-producing worms were broadly similar across hosts. This supports the interpretation that jackals can function as competent definitive hosts under certain ecological conditions.

The governance comparison is best understood from the perspective of asymmetric governance: different countries treat golden jackals as different types of political objects and the effectiveness of control is limited by both ecology and regulation. Romania’s hunting-ground–based system explicitly links monitoring and management within a regulated framework, while simultaneously documenting that expansion has continued despite management structures, implying that demographic momentum and landscape suitability can outpace control [60]. The same synthesis paper reported that in Hungary the golden jackal hunting bag increased exponentially from the mid-1990s to 2021 with an average annual growth rate of ~40% and occupancy estimated at ~86% of national territory, and suggested that intensive year-round hunting without quota restrictions has been viewed as a stabilizing mechanism in this context [60]. For Serbia, the same synthesis noted substantial territorial occupation and strong growth since the early 2000s, while linking expansion to favorable winters, anthropogenic food sources and reduced wolf presence—drivers that imply that purely lethal control may be insufficient unless it is coupled to resource management and broader landscape governance [60]. Independent evidence from legal analysis in Central Europe reinforces the broader point: the golden jackal’s status is often contested or inconsistently codified relative to its conservation status and perceived impacts, producing a patchwork of hunting and conservation obligations that can hinder coherent transboundary strategy even when ecological processes are clearly cross-border [69].

A critical and often underappreciated constraint is that hunting-based control is not simply a question of intent or effort; it is also shaped by detectability, habitat and operational conditions. Empirical evaluation of hunting efficiency in Croatia is instructive in terms of a close analogy: analyses of culled golden jackals and hunting events showed that success varies with habitat type and weather, highlighting why culling outcomes can be uneven even under active management [70]. When transposed to the Carpathian Basin context, the implication is that different strategies may sometimes be less about different policy goals than about different ecological constraints and operational realities. In areas where golden jackals rely heavily on anthropogenic subsidies, targeted management of food availability and carcass access may be as important as hunting intensity for reducing both population growth and zoonotic interface intensity.

Across both wild boar and golden jackal, the same central pattern holds: borders and land-use decisions shape permeability and permeability in turn shapes both gene flow and health dynamics. In the wild boar system, ASF response infrastructure whether behavioral (hunter biosecurity), operational (carcass search) or physical (enclosure, fencing), can restructure movement and demography in ways that may have time-lagged genetic consequences while simultaneously reshaping transmission networks [22,29,56,57]. In the golden jackal, expansion is driven by dispersal and landscape opportunity, leaves a genetic imprint of bottlenecks and admixture and is already demonstrably entangled with zoonotic hazards such as *E. multilocularis* in key parts of the Carpathian Basin and its southern periphery [61,66,67]. The shared methodological priority for the preceding sections is therefore clear: transboundary surveillance and management should be built around empirically parameterized connectivity and interface hotspots, rather than around administrative borders or national datasets considered in isolation.

A concise cross-species summary of the main pathogen/parasite occurrences discussed in this review across focal host species and regions is provided in Table 1.

## 6. Political Borders and Asymmetric Management: Biological and Epidemiological Consequences

The empirical pattern emerging from the Carpathian Basin is not simply that wildlife crosses borders, but that borders generate asymmetry—sharp, jurisdictionally imposed discontinuities in harvest regimes, feeding practices, surveillance intensity and disease-control obligations that are superimposed on populations whose biology is fundamentally transboundary. Where permeability remains high, these asymmetries can create source–sink dynamics in abundance and infection pressure, as individuals move from areas of higher density or lower mortality risk into adjacent areas managed under different rules. Where permeability is reduced, the same asymmetries can become frozen into space, producing local crowding, altered movement routes and over time, the genetic and epidemiological signatures of subdivision. In both cases, the key *One Health* consequence is that administrative fragmentation weakens the causal alignment between where management is implemented and where ecological and disease processes actually operate.

The Hungarian southern border closure illustrates this dual mechanism because it is simultaneously a physical barrier and a governance boundary. Regional analyses of the fence wave that began in 2015 document that extensive border fences were erected across South-eastern Europe in response to migrant movements, including a 175 km fence on the Hungary–Serbia border and a 136 km fence on the Hungary–Croatia border [2]. These structures were not designed as wildlife interventions, yet they function as such by reducing permeability, increasing mortality at entanglement points and displacing movement into a smaller number of leak segments where crossings remain feasible. Along the Hungary–Croatia fence, direct monitoring recorded fence-related mortality in 64 ungulates (38 red deer, 23 roe deer) and three wild boars within the first 28 months after construction, demonstrating that barrier effects manifest immediately as both welfare impacts and movement obstruction [2]. Comparable patterns were documented on the Slovenia–Croatia border, where a razor-wired fence along 178 km was associated with 21 ungulate carcasses (13 red deer, 8 roe deer) within ten months, highlighting how rapidly a state-level decision can reconfigure wildlife movement costs [1]. The most policy-relevant point, however, lies beyond direct mortality. Safner et al. [2] explicitly warn that persistent or expanding fences may fragment wildlife populations and drive genetic subdivision, including allele loss and reduced heterozygosity. This evolutionary cost of reduced dispersal is plausible for wide-ranging ungulates and wild boar in border-adjacent landscapes.

Yet fences alone do not define the risk landscape; they interact with asymmetric management on either side. In practice, borders often separate European Union (EU) and non-EU regulatory frameworks with different enforcement capacities, incentive structures and disease-control obligations. This becomes stark in the context of transboundary livestock–wildlife diseases, where domestic practices can amplify spillover pressure into wildlife and vice versa. EFSA’s risk assessment of African swine fever (ASF) for south-eastern Europe formalizes this logic through indicators that include legally defined husbandry practices and governance conditions [29]. These explicitly distinguish whether swill feeding is allowed, the extent of free-ranging pigs and home slaughtering and the prevalence of smallholders—factors that shape introduction probability and within-country spread. EFSA notes that swill feeding is prohibited in the EU (citing Commission Decision 2003/328/EC), whereas permissive or weakly enforced waste-feeding practices outside the EU can persist and elevate risk [29]. From a *One Health* perspective, this creates a structural asymmetry: a border can separate two pig-production and biosecurity regimes that generate very different pathogen pressures at the wildlife interface, while wildlife movement and informal human-mediated links (e.g., cross-border mobility, hunting tourism) continue to connect these systems [29]. In such settings, the epidemiological gradient often points from the side with higher vulnerability or poorer enforcement toward the side with stricter regulation, but the biological reality is that neither side can fully protect itself without coordinated action because the host network is shared.

Vaccination programs provide another clear example of how administrative discontinuities can imprint biological asymmetry. While ASF itself currently lacks a universally deployed wildlife vaccination strategy, analogous systems demonstrate how heterogeneous immunization landscapes emerge when programs are not aligned across borders. A retrospective analysis of classical swine fever in South-eastern Europe emphasized that mandatory vaccination was carried out in many non-EU countries, yet incomplete schemes and resource constraints contributed to continued outbreaks, with illegal swill feeding and backyard production repeatedly identified as contextual drivers [71]. Even though classical swine fever (CSF) and African swine fever (ASF) differ biologically, the governance lesson is transferable: differences in vaccination policy and implementation capacity can sustain infection reservoirs that remain epidemiologically connected to neighboring jurisdictions [71]. In wildlife, oral rabies vaccination (ORV) illustrates how a vaccination program designed for one host community can spill into others and must be managed at landscape scale. In north-eastern Romania, a historically rabies-affected region, rabies antibodies were detected in wild boar using an ELISA test [72]. The study interprets this in the context of oral rabies vaccination (ORV) campaigns targeting foxes, implying that vaccine bait distribution and uptake can intersect with omnivore behavior and cross-species contact networks. When such programs are asynchronous, discontinuous, or differentially resourced across borders, they can create spatial mosaics of immunity and exposure that do not align with wildlife movement routes, thereby undermining long-term control and complicating surveillance interpretation [72].

Asymmetric game management intensifies these dynamics because it directly shapes host density, contact rates and movement incentives. Supplemental feeding and baiting exemplify a practice whose epidemiological consequences depend on intensity and spatial configuration and which is often regulated differently across borders and even across management units within a country. In the context of ASF, an outbreak investigation in an enclosed wild boar hunting ground near the Serbia–Romania border explicitly lists wild boar supplemental feeding among human activities in forests that may contribute to indirect transmission, while also linking seasonal movement to feed sources such as corn fields and emphasizing that both human activity and landscape fragmentation modulate transmission in the border region [57]. The key asymmetry problem is straightforward: if one side of a border maintains feeding practices that elevate local density and prolong congregation at predictable points, while the neighboring side emphasizes population reduction and carcass search, the resulting density gradient and movement incentives can export risk across the border even when the recipient side applies strict control. Conversely, where border fencing reduces permeability, feeding and hunting asymmetries may be converted into localized crowding and repeated crossing attempts at limited passage points, plausibly increasing contact rates and carcass persistence in precisely the areas where surveillance is most difficult.

The golden jackal provides a parallel but distinct governance challenge, because here the biological process is a rapidly expanding mesocarnivore population that traverses administrative mosaics of legal status and culling policy. A legal analysis of Central European frameworks shows substantial contrasts not only within federal systems but also between neighboring countries and explicitly argues that this diversity of conservation and hunting laws can impede transboundary management as the golden jackal expands [69]. In practical terms, legal status is not a semantic issue: it determines whether year-round culling is permissible, whether quotas constrain removal, how damage compensation is handled and how quickly management can respond to demographic change [69]. Recent Romanian synthesis based on official records from >2100 hunting grounds characterizes Romania as operating under a quota-based hunting system tied to previous-year population estimates and argues that such an approach may be outdated given rapid expansion [60]. In the same comparative discussion, Hungary is described as having experienced an exponential increase in golden jackal hunting bag since the mid-1990s to 2021, with occupancy reaching ~86% of national territory and the cited interpretation is that intensive year-round hunting without quota restrictions has been considered the main stabilizing factor in that context [60]. Serbia, in turn, is described as undergoing strong expansion since the early 2000s from Danube-associated nuclei, consistent with the Basin-wide pattern of northward and westward spread [60]. These contrasts matter biologically because removal regimes, legal constraints and reporting requirements shape not only abundance but also dispersal pressure; asymmetry between quota-limited and quota-free systems can plausibly generate directional spillover of dispersers across borders, producing a moving front whose local density and contact behavior are partly policy-driven rather than purely ecological [60,69].

The *One Health* stakes of this asymmetry are amplified by the fact that mobile wildlife hosts connect pathogen systems regardless of jurisdiction, including zoonoses where definitive hosts (e.g., carnivores) and intermediate hosts (e.g., small mammals) respond strongly to land use and management. In Vojvodina (Serbia), golden jackals were reported with *Echinococcus multilocularis* infections in a recognized hotspot context, underlining that expanding golden jackal populations can become epidemiologically relevant alongside red foxes [66]. In south-western Hungary and the broader western Balkan region, landscape-associated patterns of *E. multilocularis* prevalence have been reported for golden jackals and foxes, reinforcing that risk is spatially structured and potentially sensitive to cross-border differences in carnivore density, hunting pressure and access to anthropogenic food subsidies [67]. When such zoonotic hazards are embedded in populations whose legal status and management intensity differ across borders, the epidemiological system becomes patch-managed despite being biologically continuous.

The work, therefore, is not a generic claim that wildlife does not know borders, but a more operational argument: administrative fragmentation creates mismatched management fields across a connected host landscape through three linked mechanisms (altered permeability, asymmetric host density/contact structure and uneven surveillance and response capacity) and this mismatch increases both the probability and the consequences of disease emergence and persistence. Border fences may temporarily reduce certain movement routes, but evidence from South-eastern Europe indicates that they impose nontrivial ecological and welfare costs and may also generate long-term genetic subdivision risks if they persist [1,2]. Meanwhile, differences in husbandry regulation (e.g., swill feeding prohibition in the EU), surveillance infrastructure, vaccination program implementation and wildlife management strategies (feeding, harvest regimes, legal status of expanding species) can produce transboundary gradients in density and infection pressure that are not resolvable within national borders [29,57,60,69,71,72].

## 7. Conclusions and Carpathian Basin-Scale One Health Priorities

This review supports a simple but consequential conclusion: the Carpathian Basin functions as a biologically connected wildlife system, whereas management and surveillance remain primarily organized along national administrative lines. Across focal taxa, this mismatch creates a recurring *One Health* problem: movement supports both demographic/genetic resilience and pathogen circulation, while differences in policy, surveillance intensity and intervention practice generate uneven risks across a shared ecological space [1,2,3,4].

Genetic and landscape evidence further indicates that connectivity is substantial at the basin scale but spatially heterogeneous and that infrastructure, urban matrices and fencing can reshape permeability in ways that matter for both epidemiology and long-term population structure [3,4,22]. Accordingly, transboundary wildlife health planning in the region should be designed for a biologically continuous system rather than inferred from administrative boundaries alone.

The examples summarized in this review—especially ASF in wild boar systems and the expansion of the golden jackal with associated zoonotic interfaces—show that fragmented governance can delay coherent response, complicate risk interpretation and produce avoidable asymmetries in prevention and control [29,56,60,66,67,69]. The central implication is not that all countries should apply identical measures, but that surveillance logic, reporting standards and interpretation frameworks must be interoperable across borders in a biologically continuous system.

A feasible basin-scale *One Health* response should therefore prioritize three elements. First, harmonized wildlife health surveillance, combining standardized passive surveillance (including carcass reporting/search protocols in high-risk mobile hosts) with targeted active surveillance in priority host–pathogen systems [29,56]. Second, landscape-aware risk assessments, explicitly accounting for permeability, barriers, transport corridors and habitat structure when defining surveillance zones and interpreting transboundary spread [11,22,38,41]. Third, shared genetic infrastructure and data standards, so connectivity, barrier effects and demographic change can be tracked comparably through time and used in outbreak attribution and preparedness across the region [3,20,21,44,62]. The available literature was highly heterogeneous in study designs, markers/diagnostics and reported outcomes, which precluded a quantitative meta-analysis and supports interpretation primarily at the level of narrative synthesis. We did not apply a formal risk-of-bias tool or certainty assessment framework (e.g., GRADE), and therefore the strength of evidence is discussed qualitatively. In addition, records without a DOI were excluded during screening, which may have led to the omission of some older or regionally disseminated studies. We also acknowledge likely geographic publication bias across the Carpathian Basin, as study availability may partly reflect country-level differences in surveillance intensity, research capacity and publication practices rather than the true absence of hosts or pathogens.

## Figures and Tables

**Table 1:** Regional occurrence of major pathogens and parasites in the focal wildlife species reviewed.

Host Species	Main Agents	Epidemiological Interpretation	Regions	References
Wild boar	ASF; *Trichinella* spp.	Major transboundary host in ASF dynamics; carcass-mediated persistence and barrier effects are emphasized	Serbia; Serbia–Bosnia and Herzegovina; broader Carpathian region	[16,29,30,52,53,54,55,56,57]
Red deer	Tick-borne disease relevance; CWD; mycobacteria/bTB	Connectivity and aggregation may shape pathogen spread; CWD preparedness is a regional priority	Slovenia; Central Europe; Europe	[35,40,49,50]
Roe deer	Tick-borne microorganisms; *A. phagocytophilum* ecotypes	Important tick-supporting host; local structure may contribute to focal zoonotic hazard	Slovakia; Central Europe; Slovenia; Hungary	[11,38,39,40,41,42]
Fallow deer	*F. magna*; bacterial communities; mycobacteria/bTB	Managed/fenced populations may amplify transmission and cross-border parasite exchange	Serbia; Hungary/Croatia Danube context; Romania; Central Europe	[46,47,48,49,50,51]
Golden jackal	*E. multilocularis*; *D. immitis*; *Trichinella* spp.	Range expansion may redistribute zoonotic risk and parasite communities	Serbia; Vojvodina; south-western Hungary/western Balkans	[64,65,66,67,68]

## Data Availability

No new data were created or analyzed in this study. Data sharing is not applicable to this article.

## Supplementary Materials

The following supporting information can be downloaded at: https://www.mdpi.com/article/10.3390/vetsci13030302/s1, Table S1: PRISMA_2020_checklist.
