# Supplementary material for: Without Borders? The Impact of Political Barriers and Land Use on the Animal Health Dynamics and Genetic Structures of Large Game Species in the Carpathian Basin and Surrounding Regions—A Systematic Review
Source: Vet Sci. 2026 Mar 23;13(3):302. doi: 10.3390/vetsci13030302 (PMC13030641; doi:10.3390/vetsci13030302)
Supplement: Supplementary file 1 [file vetsci-13-00302-s001.zip › vetsci-4207589-supplementary.pdf]

PRISMA

PRISMA 2020 Checklist

Section and Topic	Item #	Checklist item	Location where item is reported
**TITLE**			
Title	1	Identify the report as a systematic review.	Title page – manuscript title (include the phrase “Systematic Review”).
**ABSTRACT**			
Abstract	2	See the PRISMA 2020 for Abstracts checklist.	Abstract.
**INTRODUCTION**			
Rationale	3	Describe the rationale for the review in the context of existing knowledge.	Section 1 (Introduction) – rationale/background.
Objectives	4	Provide an explicit statement of the objective(s) or question(s) the review addresses.	Section 1 (Introduction) – objectives/aims (final paragraph).
**METHODS**			
Eligibility criteria	5	Specify the inclusion and exclusion criteria for the review and how studies were grouped for the syntheses.	Section 2 (Literature search and study selection) – inclusion/exclusion criteria and grouping.
Information sources	6	Specify all databases, registers, websites, organisations, reference lists and other sources searched or consulted to identify studies. Specify the date when each source was last searched or consulted.	Section 2 – information sources (databases) and search dates.
Search strategy	7	Present the full search strategies for all databases, registers and websites, including any filters and limits used.	Section 2 – search strategy overview; full search strings provided in Supplementary Table S1 (to upload).
Selection process	8	Specify the methods used to decide whether a study met the inclusion criteria of the review, including how many reviewers screened each record and each report retrieved, whether they worked independently, and if applicable, details of automation tools used in the process.	Section 2 – screening process (title/abstract then full text); add a sentence on number of reviewers and how disagreements were resolved.
Data collection process	9	Specify the methods used to collect data from reports, including how many reviewers collected data from each report, whether they worked independently, any processes for obtaining or confirming data from study investigators, and if applicable, details of automation tools used in the process.	Section 2 – data extraction process; add a sentence on who extracted and whether it was cross-checked.
Data items	10a	List and define all outcomes for which data were sought. Specify whether all results that were compatible with each outcome domain in each study were sought (e.g. for all measures, time points, analyses), and if not, the methods used to decide which results to collect.	Section 2 – data items/outcomes extracted (genetic diversity/structure/phylogeography; health/One Health outcomes where applicable).
	10b	List and define all other variables for which data were sought (e.g. participant and intervention characteristics, funding sources). Describe any assumptions made about any missing or unclear information.	Section 2 – other variables extracted (species, country/region, marker/assay, sampling/study design, key methods).
Study risk of bias assessment	11	Specify the methods used to assess risk of bias in the included studies, including details of the tool(s) used, how many reviewers assessed each study and whether they worked independently, and if applicable, details of automation tools used in the process.	To add in Section 2 (or Section 7 Limitations): no formal risk-of-bias tool applied; qualitative appraisal due to heterogeneity.

PRISMA

PRISMA 2020 Checklist

Section and Topic	Item #	Checklist item	Location where item is reported
Effect measures	12	Specify for each outcome the effect measure(s) (e.g. risk ratio, mean difference) used in the synthesis or presentation of results.	Not applicable (no quantitative synthesis/meta-analysis planned or performed); Section 2 (narrative synthesis).
Synthesis methods	13a	Describe the processes used to decide which studies were eligible for each synthesis (e.g. tabulating the study intervention characteristics and comparing against the planned groups for each synthesis (item #5)).	Section 2 – narrative synthesis methods and grouping (by species/theme/region).
	13b	Describe any methods required to prepare the data for presentation or synthesis, such as handling of missing summary statistics, or data conversions.	Not applicable (no meta-analysis; no quantitative data transformations).
	13c	Describe any methods used to tabulate or visually display results of individual studies and syntheses.	Sections 3–6 – narrative presentation of results; Figure 1 for study flow.
	13d	Describe any methods used to synthesize results and provide a rationale for the choice(s). If meta-analysis was performed, describe the model(s), method(s) to identify the presence and extent of statistical heterogeneity, and software package(s) used.	Sections 3–6 (synthesis by theme/species) and Section 7 (Conclusions).
	13e	Describe any methods used to explore possible causes of heterogeneity among study results (e.g. subgroup analysis, meta-regression).	Not applicable (no quantitative heterogeneity assessment; no meta-analysis).
	13f	Describe any sensitivity analyses conducted to assess robustness of the synthesized results.	Not applicable (no sensitivity analyses; no meta-analysis).
Reporting bias assessment	14	Describe any methods used to assess risk of bias due to missing results in a synthesis (arising from reporting biases).	Not assessed; to add a brief statement in Section 2 or Section 7 (Limitations).
Certainty assessment	15	Describe any methods used to assess certainty (or confidence) in the body of evidence for an outcome.	Not assessed (no GRADE/certainty framework); to add a brief statement in Section 7 (Limitations).
**RESULTS**			
Study selection	16a	Describe the results of the search and selection process, from the number of records identified in the search to the number of studies included in the review, ideally using a flow diagram.	Section 2 (methods summary of selection) and Figure 1 (PRISMA 2020 flow diagram).
	16b	Cite studies that might appear to meet the inclusion criteria, but which were excluded, and explain why they were excluded.	Figure 1 (full-text exclusions with reasons).
Study characteristics	17	Cite each included study and present its characteristics.	Sections 3–6 – characteristics of included studies described in-text (no summary table currently).
Risk of bias in studies	18	Present assessments of risk of bias for each included study.	Not assessed (see Item 11).
Results of individual studies	19	For all outcomes, present, for each study: (a) summary statistics for each group (where appropriate) and (b) an effect estimate and its precision (e.g. confidence/credible interval), ideally using structured tables or plots.	Sections 3–6 – results of individual studies within species/thematic subsections.
Results of syntheses	20a	For each synthesis, briefly summarise the characteristics and risk of bias among contributing studies.	Sections 3–6 (narrative syntheses) and Section 7 (Conclusions).
	20b	Present results of all statistical syntheses conducted. If meta-analysis was done, present for each the summary estimate	Not applicable (no meta-analysis).

PRISMA 2020 Checklist

Section and Topic	Item #	Checklist item	Location where item is reported
		and its precision (e.g. confidence/credible interval) and measures of statistical heterogeneity. If comparing groups, describe the direction of the effect.	
	20c	Present results of all investigations of possible causes of heterogeneity among study results.	Not applicable (no meta-analysis; no heterogeneity exploration).
	20d	Present results of all sensitivity analyses conducted to assess the robustness of the synthesized results.	Not applicable (no sensitivity analyses).
Reporting biases	21	Present assessments of risk of bias due to missing results (arising from reporting biases) for each synthesis assessed.	Not assessed.
Certainty of evidence	22	Present assessments of certainty (or confidence) in the body of evidence for each outcome assessed.	Not assessed.
**DISCUSSION**			
Discussion	23a	Provide a general interpretation of the results in the context of other evidence.	Section 7 (Conclusions and One Health priorities).
	23b	Discuss any limitations of the evidence included in the review.	To add in Section 7 – limitations of included evidence (heterogeneity, uneven geographic coverage, etc.).
	23c	Discuss any limitations of the review processes used.	To add in Section 7 – limitations of review processes (e.g., DOI-based retrieval excluding non-DOI records; retrospective registration).
	23d	Discuss implications of the results for practice, policy, and future research.	Section 7 – implications for practice/policy and future research (Basin-scale One Health monitoring/data-sharing).
**OTHER INFORMATION**			
Registration and protocol	24a	Provide registration information for the review, including register name and registration number, or state that the review was not registered.	Section 2 (end of Methods): statement that the review was not registered and no prospectively registered protocol was available.
	24b	Indicate where the review protocol can be accessed, or state that a protocol was not prepared.	Not applicable (no protocol prepared / no public protocol available); stated in Section 2.
	24c	Describe and explain any amendments to information provided at registration or in the protocol.	Not applicable (no protocol amendments).
Support	25	Describe sources of financial or non-financial support for the review, and the role of the funders or sponsors in the review.	Funding statement and Acknowledgments.
Competing interests	26	Declare any competing interests of review authors.	Conflicts of Interest statement.
Availability of data, code and other materials	27	Report which of the following are publicly available and where they can be found: template data collection forms; data extracted from included studies; data used for all analyses; analytic code; any other materials used in the review.	Data Availability Statement; Supplementary materials (search strings, PRISMA checklist) where applicable.

From: Page MJ, McKenzie JE, Bossuyt PM, Boutron I, Hoffmann TC, Mulrow CD, et al. The PRISMA 2020 statement: an updated guideline for reporting systematic reviews. BMJ 2021;372:n71. doi: 10.1136/bmj.n71.
This work is licensed under CC BY 4.0. To view a copy of this license, visit https://creativecommons.org/licenses/by/4.0/
